# Dietary L-Arginine Supplementation Affects the Skeletal Longissimus Muscle Proteome in Finishing Pigs

**DOI:** 10.1371/journal.pone.0117294

**Published:** 2015-01-30

**Authors:** Xianyong Ma, Chuntian Zheng, Youjun Hu, Li Wang, Xuefen Yang, Zongyong Jiang

**Affiliations:** 1 Institute of Animal Science; Guangdong Academy of Agricultural Sciences, Guangzhou, China; 2 State Key Laboratory of Livestock and Poultry Breeding, Guangzhou, China; 3 Key Laboratory of Animal Nutrition and Feed Science in South China, Ministry of Agriculture, Guangzhou, China; 4 Guangdong Public Laboratory of Animal Breeding and Nutrition, Guangzhou, China; 5 Guangdong Key Laboratory of Animal Breeding and Nutrition, Guangzhou, China; National Institute of Agronomic Research, FRANCE

## Abstract

Forty-eight Duroc x Landrace x Large White gilts were used to determine the relationship between proteome changes of longissimus muscle and intramuscular fat (IMF) content in arginine-supplemented pigs. Beginning at 60 kg BW, pigs were fed a corn- and soybean meal-based diet supplemented or not with 1% L-arginine until they reached a BW of 100 kg. Supplementation with 1% L-arginine did not affect the growth performance or carcass traits, while it increased IMF content by 32% (P < 0.01), it also decreased the drip loss at 48 h post-mortem and the b* meat color value at 24 h post-mortem; supplementation with 1% dietary L-arginine did not change the proportion of SFA and MUFA in muscle lipids. The proteome changes in longissimus muscle between the control and supplemented pigs showed that L-arginine significantly influenced the abundance of proteins related to energy metabolism, fiber type and structure. The increase in IMF content was positively correlated with the increased abundance of slow twitch troponin I (TNNI1) protein and negatively correlated with myosin heavy chain IIb (MyHC IIb) protein content. It is suggested that the proteome changes in longissimus muscle contributed to the greater IMF content in L-arginine supplemented pigs.

## Introduction

Dietary L-arginine has been reported to reduce body fat and increase brown adipose fat in several species [1–2]. In pigs, L-arginine also has been reported to reduce backfat thickness, and increase both marbling score [3–4] and intramuscular fat (IMF) content [4–5]. Increasing IMF content is important for the swine industry because it is positively correlated with pork quality, especially tenderness [6] and juiciness [7].

Although a number of biochemical and molecular mechanisms have been proposed to explain the roles for L-arginine in metabolism of energy substrates [8–10], it is not clear how L-arginine increases IMF content. Development of adipose tissue is positively associated with expression of the *peroxisome proliferators-activated receptor γ* (*PPARγ*) gene [11]. Previous work from this laboratory found that dietary supplementation with 1% L-arginine increased expression of adipocyte-specific *PPARγ* in pig muscle [5]. Arginine supplementation also affected the composition of fatty acids in rat [12], porcine [13], and bovine [14] white adipose tissues, as well as expression of genes related to fat metabolism in this tissue [15]. Singh et al. [16] reported that altered *PPARγ* expression inhibited the expression of myogenic proteins and the formation of myotubes, while myofibril proteins such as actin and PDZ protein decreased during adipogenesis [16–17]. The characteristics of slow-twitch and fast-twitch fibers in skeletal muscle play a key role in meat quality [18] and slow-twitch muscle contains more IMF than does fast-twitch muscle [19]. L-Arginine supplementation also up-regulated the expression of lipogenic genes, such as those encoding lipoprotein lipase (LPL) and acetyl-CoA carboxylase (ACC) alpha [20]. On the basis of the foregoing information, it was hypothesized that L-arginine might influence IMF by its effects on structure-related proteins, fiber type transformation and energy metabolism in muscle.

Proteomics technology allows simultaneously examining the differential expression of a large number of proteins [21–23] and has been widely used to compare many proteins related to meat quality attributes [24–26] and nutrient metabolism [27–28]. The objective of the present study was to identify proteins that are differentially expressed in the longissimus muscle of finishing pigs supplemented with 1% arginine and to further explain the mechanisms of IMF content changed with supplementation of arginine.

## Materials and Methods

### Pigs and diets

Forty-eight Duroc × Landrace × Large White gilts (free of the halothane gene) of approximately 60 kg body weight (BW) were assigned to 2 treatments, each consisting of 6 replicates (pens) of 4 pigs. All pigs were housed in the animal facilities of the Institute of Animal Science in the Guangdong Academy of Agricultural Sciences. Pigs were fed a corn- and soybean meal-based diet (Table 1), supplemented with 1% L-arginine (98% purity) or with iso-nitrogenous amounts of L-Alanine as controls, as described by Kim and Wu et al.[29]. Both amino acids were purchased from Guangzhou Weijian Medicine and Healthy Foods Import—Export Company (Guangdong, China).

**Table 1:** Formulation and nutrient content of diets.

Diet ingredients (% by weight)	Control	Arginine Supplemented
Corn meal	72.7	73.1
Soybean meal	17.6	17.6
Wheat bran	3	3
Rape meal	4	4
Alanine	1.4	0
Arginine	0	1
Premix*	1	1
L-lysine HCl	0.12	0.12
Limestone	1	1
Salt	0.3	0.3
Dicalcium phosphate	0.3	0.3
Total	101.42	101.42
Digestible energy (Mcal/kg)	3.23	3.23
CP (%)	15.51	15.51
Lys (%)	0.80	0.80
Met (%)	0.24	0.24
Met+Cys (%)	0.51	0.51
Thr (%)	0.58	0.58
Trp (%)	0.17	0.17
Arg(%)	0.96	1.96
Valine(%)	0.71	0.71
Ca (%）	0.55	0.55
Total P (%)	0.42	0.42
Available P (%)	0.19	0.19
Na (%)	0.15	0.15
Cl (%)	0.24	0.24

*Premix contained 1750 IU/kg vitamin A, 220 IU/kg vitamin D3, 3 IU/kg vitamin E, 0.55 mg/kg vitamin K3, 0.25 mg/kg vitamin B1, 1.0 mg/kg vitamin B2, 0.7 mg/kg vitamin B6, 3 μg/kg vitamin B12, 4 mg/kg niacin, 1.6 mg/kg calcium pantothenate, 0.1 mg/kg folic acid, 7 μg/kg biotin, 0.08 g/kg choline chloride, 6.5 mg/kg Mn, 15mg/kg Fe, 15 mg/kg Zn, 1.5 mg/kg copper (Cu), 0.07 mg/kg iodine (I_2_), 0.03 mg/kg selenium (Se), and 1 g/kg sodium chloride (NaCl).

### Feeding and slaughter procedure

Pigs were fed until they reached 100 kg BW. The amount of feed consumed was recorded for each replicate to determine average daily gain (ADG), average daily feed intake (ADFI) and gain feed ratio (G:F). At the end of the experiment, 14 h after the last feeding, pigs were bled immediately before electro-stunning and exsanguination. Experiments involving slaughtering and transport procedures on live animals were carried out in accordance with the Chinese guidelines for the use of experimental animals and animal welfare [30] and approved by The International Cooperation Committee of Animal Welfare (ICCAW, established in Beijing, China).

### Sample collection

Blood was collected from the anterior vena cava using vacuum tubes (no anticoagulant), allowed to clot at room temperature for 120 min, centrifuged for 5 min at 3,000 × *g* at 4°C and the serum was stored at -20°C following slaughter, samples of longissimus muscle over the ninth to tenth ribs were immediately obtained and frozen in liquid N_2_ for measurement of IMF content, fatty acid composition, enzyme activities and proteomics analysis. Fresh samples of longissimus muscle (1 cm^3^) were fixed in 4% paraformaldehyde in PBS (pH 7.3). Back fat thickness was measured on the midline over the first, 10th and last rib and cross-sectional area of the longissimus muscle was measured at the junction of thoracic and lumbar vertebrae, by tracing onto sulfate paper followed by planimetry.

### Measurement of biochemical variables in plasma

Plasma concentrations of HDL (high density lipoprotein), LDL (low density lipoprotein), glucose, cholesterol and triglycerides were determined using an automatic analyzer (cx5, Beckman Coulter INC, Brea, CA) and the concentration of leptin was determined using an ELISA kit (Luyu Bioengineering, Shanghai, China).

### Meat quality measurements

Muscle samples were taken at the last thoracic vertebra and their pH values were recorded at 45 min, 24 h and 48 h postmortem using a pH meter (HI 8242C, Beijing Hanna Instruments Science & Technology, Beijing, China). Drip loss was estimated according to the method described by Ma et al. [5]. Meat color CIE LAB values (L*, relative lightness; a*, relative redness; b*, relative yellowness) were determined on the surface of the meat sample after the sample was cut and exposed to air for 45 min (a complete cross section at the thoracolumbar junction, approximately 2 cm thickness) with a colorimeter (CR-410, Minolta, Suita-shi, Osaka, Japan) as described by Mason et al. [31]. Shear force was measured using an Instron Universal Mechanical Machine (Instron model 4411, Instron, Canton) as described by Ma et al. [5]. The marbling of meat samples (thoraco-lumbar vertebra section) was assessed by a marbling testing standard [32] after being stored at 4°C for 24 h.

### Measurement of intramuscular fat content

The longissimus muscle samples were lyophilized and ground to powders. The IMF content was measured by petroleum ether (30 to 60°C boiling point) extraction using the Soxtec 2055 fat extraction system (Foss Tecator AB, Sweden), according to the Association of Official Analytical Chemists method [33].

### Fatty acid composition analysis

Samples of longissimus muscle were taken from -80°C refrigerator and crushed and ground to power, then were saponified and methylated using procedures, essentially as described by Azain [34]. Standard fatty acid methyl esters (FAME) were purchased from Sigma (Sigma-Aldrich, St Louis, MO). The FAME were separated on a gas chromatograph (Thermo Electron Co., San Jose, CA) equipped with a capillary column (30 m × 0.25 mm; 0.25 μm film thickness). Injector and detector temperatures were 250°C and the oven was held at 160°C for 3 min then increased to 190°C (at 5°C/min) to 210°C (at 2°C/min), and finally to 250°C at 15°C/min). Each sample of FAME was injected (1 μL) into the split injection port (30:1 split ratio). The flow rate of helium was 1 mL/min. Individual FAME were identified from their retention times and was verified by mass spectrometry at 70 eV ionization potential and a scan range of 45 to 625 mass-to-charge ratio.

### Diameter and the density of intramuscular adipocytes

Fixed tissues were embedded in paraffin, sectioned at 5 μm, dewaxed, and stained with hematoxylin and eosin (Beijing Biosynthesis Biotechnology Co., Ltd., Beijing, China). The sections were viewed at 10x magnification using Motic BA400 microscope and the diameter and density of the intramuscular adipocytes were determined with Motic image software (Motic-Optic Industrial Group Co. Ltd., Xiamen, China).

### Proteome analysis

**Sample preparation.** Frozen muscle samples (1 pooled sample from the 4 pigs per replicate) were crushed and homogenized in 3 mL lysis buffer consisting of 2 M thiourea, 8 M urea, 65 mM 3-((3-cholamidopropyl) dimethylammonio)-1-propane sulfonate, 60 mM dithiothreitol, 0.5 mM phenylmethanesulfonyl fluoride and 40 mM Tris (pH 8.6) by sonication for 3 min in an ice bath, using 6 pulses of 30s at 50-W. The homogenate was centrifuged at 15,000 × *g* for 45 min at 4°C. The non-fat supernatant was re-centrifuged at 40,000 × *g* for 45 min at 4°C. Protein concentration was determined using a protein assay kit (Bio-Rad, Hercules, CA), with bovine serum albumin as standard [35].

**Two-dimensional gel electrophoresis (2-DE).** 2-DE was performed according to Xu et al [36] with some modification. Briefly, 200 μg protein of each sample was applied to 17-cm immobilized pH gradient (IPG) strips (3–10 nonlinear, Bio-Rad). Samples were applied during overnight rehydration with the lysis buffer (2M thiourea, 8M urea, 65mM 3-((3-cholamidopropyl) dimethylammonio)-1-propane sulfonate, 60 mM dithiothreitol and 40 mM Tris (pH 8.6)). After that, the IPG strips were removed to a focusing tray and any proteins that had not been absorbed into the gel strip were removed. Isoelectric focusing (IEF) was conducted using a Protean IEF Cell (Bio-Rad) according to the following IEF parameters: rapid ramping to 100 V, 300 V, 500 V, 800 V and 1,000 V was performed to desalt for 1 h, 1.5 h, 2.5 h, 2.5 h and 2.5 h, respectively, linear ramping to 8,000 V for 3 h, rapid ramping to 10,000 V and a constant of 10,000 V until approximately 65 kVh was reached. After IEF, IPG strips were incubated for 20 min with 10 ml equilibration solution (50 mM Tris-HCl (pH 8.8), 6 M urea, 2% (m/v) sodium dodecyl sulfate (SDS), 20% (v/v) glycerol and 1% (m/v) DTT). In the second equilibration solution, 1% (m/v) DTT was replaced with 2.5% (m/v) iodoacetamide. The strip was immersed for 1 s in the buffer containing 0.3% (m/v) Tris-HCl, 1.44% (m/v) glycine and 0.1% (m/v) SDS to remove the excess equilibration buffer on the surface of strip. The second dimension SDS-PAGE was carried out in a Protean II xi Cell (Bio-Rad) with 12% polyacrylamide gels (36.5:1 ratio of acrylamide to bisacrylamide). Electrophoresis was performed at 12°C used 12 mA/gel for 30 min, then transferred to 25 mA until bromophenol blue reached the end of gel.

Gels were stained with silver nitrate according to the protocol: The gel was soak in fixing solution containing 50% MeOH, 12%Hac and 0.05% formalin for 30 min, then washed three times in distilled water for 5 min each time; Sensitizing solution containing 0.02% Na2S2O3 was added and leave shaking for 30 min, then removed and the gel was washed three times in distilled water for 5 min each time; Silver solution containing 0.2% AgNO3 and 0.076% formalin was added and leave shaking for 20 min, then removed and the gel was rinsed twice in distilled water for 1 min each time; Developing solution containing 6% NaCO3, 0.05% formalin and 0.0004% Na2S2O3 was added and leave shaking for 2–5 min, then removed. Stop solution containing 50% MeOH, 12%Hac was added and leave shaking for 10 min, then remove and the gel was washed three times in distilled water for 5 min each time.

**Image analysis.** Destained gels were immediately scanned (imaging system, Versadoc Model 3000, Bio-Rad, USA) and analyzed using 2D image analysis software (PDQuest, Bio-Rad, USA) according to the manufacturer’s instruction. Molecular weights were estimated by application of marker proteins (Low Molecular Weight SDS Calibration Kit for SDS electrophoresis, Amersham Pharmacia Bio-tech), and pI was estimated by assuming linear distribution of pI over the whole range of the Immobiline DryStrip pH 3–10. Sample gels, 1 from each of the 6 replicates of control and L-arginine-supplemented pigs were obtained then the best 3 of each 6 were selected on the basis of protein spot pattern and number. Statistical analysis was done by student t-test, as provided by the PDQuest software.

**Protein digestion and HPLC-MS/MS analysis.** Targeted protein spots were excised and digested with trypsin, followed by the HPLC-MS/MS analysis, as described by Han et al.[37]with some modifications. Briefly, spots of interest were excised, and the proteins were subjected to in-gel tryptic digestion and peptide extraction using a Montage In-Gel Digest 96 ZP kit (Millipore, Billerica, USA). The peptides were analyzed using a matrix-assisted laser desorption ionization time-of-ﬂight (MALDI-TOF) mass spectrometer 4800 Proteomics Analyzer (Applied Biosystems). The 4700 calibration mixtures (Applied Biosystems) were used to calibrate the spectrum to a mass tolerance within 0.1 Da. Internal calibration was performed using peptides resulting from auto-digestion of porcine trypsin. Proteins were identified from their peptide mass fingerprint by searching the National Center for Biotechnology Information (NCBI) protein sequence database using the MASCOT and PROFOUND softwares (http://www.matrixscience.com and http://prowl.rockefeller.edu). All of the automatic data analysis and database searching were fulfiled by the GPS Explorer software (version 3.6, Applied Biosystems). The identification had a statistically significant (P<0.05) protein score (based on combined mass and mass/mass spectra) and best ion score (based on mass/mass spectra). Protein redundancy that appeared in the database under different names and accession numbers was eliminated. If one protein spot was identified as more than one protein, the single protein member with the high best protein score (top rank) was selected from the multi-protein family. The molecular weight and pI values of most proteins were consistent with the gel regions from which the spots were excised.

**Western-blotting.** Pools of muscle proteins (40 μg, 1 for each replicate) were separated by SDS-PAGE according to Nirmalan et al. [38] and proteins were electro-transferred to PVDF membranes at 250 mA for 90 min. Membranes were blocked overnight at 4°C with 5% non-fat milk in TBST (10 mM Tris-HCl, pH 8.0, 150 mM NaCl, 0.1% Tween 20). All primary antibodies were raised in rabbits and were suitable for detecting specific porcine proteins: Troponin I type 1(slow skeletal), NBP1–56641, NOVUS Biologicals (Littleton, CO); MyH IIb, ab110317; creatine kinase, AB126244; and GADPH, AB9485, ABCAM Biotechnology (Cambridge, UK). Each was used at 1:250 dilutions in blocking buffer for 2 h at room temperature. Membranes were washed 3 times, each for 10 min, with 10 mL TBST then incubated for 1 h in blocking buffer containing horseradish peroxidase-labeled (HRP) anti-rabbit secondary antibody (ABCAM Biotechnology, Cambridge, UK) diluted 1:5,000. After three 10-min washes, immune-reactive proteins were visualized using a chemiluminescent HRP substrate (Millipore, Billerica, MA) and a VersaDoc imaging system (Bio-Rad). The band densities were calculated by Quantity One software and normalized to the density of GADPH (Santa Cruz Biotechnology, Inc., Santa Cruz, CA). All the proteins were indentified in the same SDS-PAGE gel and transferred to the same PVDF membrane, then striped and probed respectively with their antibody. The Western-blot analyses were performed in triplicate.

### Statistical analysis

Data for growth performance, meat quality, fatty acid composition, blood variables, diameter and density of adipocytes were analyzed by independent-samples t-test methods using SPSS11.5 (SPSS Inc., Chicago, IL, USA). Results are expressed as mean ±SEM. Data for the scanning two-dimensional gel were analyzed using Image Master 2D image analysis software (PDQuest, Bio-Rad, USA). Proteins were identified using the MASCOT and PROFOUND softwares (http://www.matrixscience.com and http://prowl.rockefeller.edu) and the GPS Explorer software (version 3.6, Applied Biosystems). Data for Western-blot were calculated by Quantity One software and analyzed by independent-samples t-test methods using SPSS11.5 (SPSS Inc., Chicago, IL, USA). *P*-values < 0.05 were considered to be significant and *P*-values < 0.01 were considered to be highly significant for all the data in this manuscript. In addition, all data and related metadata were deposited in the file at Institute of Animal Science, Guangdong Academy of Agricultural Sciences and available for share.

## Results

### 1 Effect of dietary L-arginine on growth performance and carcass traits of pigs

The growth performance and carcass traits of the pigs are summarized in Table 2. Supplementation with 1% L-arginine did not affect (*P* > 0.05) ADG, ADFI, G:F, loin eye area or back fat thickness.

**Table 2:** Effect of dietary L-arginine supplementation on growth performance

Variable		Control	1% arginine
ADG (kg)		0.79 ± 0.03	0.77 ± 0.02
ADFI (kg)		2.69 ± 0.10	2.69 ± 0.05
F:G		3.43 ± 0.08	3.50 ± 0.07
Loin eye area (mm^2^)		5375 ± 258	5459 ± 87
Backfat depth (cm)	First rib	4.00 ± 0.12	3.88 ± 0.34
	Tenth rib	2.07 ± 0.20	1.99 ± 0.14
	Last rib	1.27 ± 0.06	1.20 ± 0.09

Data are means ± SEM, n = 24. There were no significant effects of supplementation (*P* > 0.05).

ADG average daily gain, ADFI average daily feed intake, F:G feed to gain ratio.

### 2. Effect of dietary L-arginine on meat quality traits of pigs

As shown in Table 3, the pH (at 45 min and 24 h post-mortem), drip loss (at 24 h post-mortem), meat color traits (a*, b*, L value at 45 min and a* and L value at 24 h post-mortem) and shear force of meat did not differ (*P* > 0.05) between the control and 1% L-arginine supplemented pigs. The drip loss at 48 h post-mortem was lower (*P* < 0.05) in pigs supplemented with 1% L-arginine compared with the control pigs. The IMF content was 32.1% higher (*P* < 0.01) in pigs supplemented with 1% L-arginine than in the controls.

**Table 3:** Effect of dietary L-arginine supplementation on indices of meat quality.

Variable			Control	1% arginine
pH	45 min		6.50 ± 0.06	6.57 ± 0.06
	24 h		5.57 ± 0.06	5.65 ± 0.06
Drip loss (%)		24 h	1.21 ± 0.05	1.02 ± 0.07
		48 h	2.32 ± 0.06	2.04 ± 0.10*
Meat color	45 min	a*	15.57 ± 0.23	15.74 ± 0.25
		b*	2.70 ± 0.20	2.60 ± 0.19
		L	45.85 ± 0.51	45.74 ± 0.48
	24 h	a*	18.03 ± 0.29	18.40 ± 0.28
		b*	7.36 ± 0.36	2.63 ± 0.47*
		L	52.24 ± 0.44	51.71 ± 1.17
Shear force（Nf）			46.03 ± 1.86	43.04 ± 1.21
Intramuscular fat (%)			3.96 ± 0.41	5.23 ± 0.35**

Data are means ± SEM, n = 24. Within a row, means with * indicate a significant difference (*P* < 0.05), means with ** indicate a significant difference (P < 0.01).

L, lightness; a*, redness; b*, yellowness.

### 3. Effect of dietary L-arginine on fatty acid composition and proportion in longissimus muscle

Dietary supplementation with 1% arginine did not affect the relative contents of total saturated fatty acid (SFA) and mono-unsaturated fatty acid (MUFA) in lipids extracted from muscle (Table 4), but it did increase the relative contents of C20:1(*P* < 0.01), C22:6 and total polyunsaturated fatty acid (PUFA) (*P* < 0.05).

**Table 4:** Effect of L-arginine on fatty acid composition in longissimus muscle.

Fatty acid	Control	1% arginine
C14:0	1.71 ± 0.19	1.65 ± 0.15
C15:0	0.03 ± 0.008	0.03± 0.008
C16:0	24.50 ± 0.90	23.12 ± 0.78
C16:1	4.25 ± 0.46	4.32 ± 0.71
C17:0	0.19 ± 0.04	0.17 ± 0.04
C17:1	0.25 ± 0.05	0.23 ± 0.04
C18:0	14.56 ± 0.49	15.46 ± 0.96
C19:0	0.06 ± 0.01	0.05 ± 0.01
C18:1	44.97 ± 0.78	44.64 ± 1.77
C18:2	7.08 ± 0.37	7.76 ± 0.11
C18:3	0.27 ± 0.04	0.31 ± 0.02
C20:0	0.24 ± 0.04	0.27 ± 0.04
C20:1	0.86 ± 0.08	1.11 ± 0.10**
C20:2	0.36 ± 0.06	0.33 ± 0.03
C20:3	0.10 ± 0.02	0.09 ± 0.02
C20:4	0.40 ± 0.03	0.39± 0.05
C22:6	0.15 ± 0.06	0.39± 0.06*
SFA	41.23± 1.64	40.75 ± 1.98
MUFA	50.33 ± 1.37	50.30 ± 2.62
PUFA	8.36 ± 0.58	9.27 ± 0.30*

Data are means of fatty acid percentages ± SEM, n = 24.

Within a row, means with * indicate a significant difference (*P* < 0.05), means with ** indicate a significant difference (P < 0.01).

SFA, saturated fatty acids; MUFA, monounsaturated fatty acids; PUFA, polyunsaturated fatty acids.

### 4. Effect of dietary L-arginine on the diameter and density of intramuscular fat cells in longissimus muscle

Supplementation with 1% L-arginine did not result in significant changes in the diameter or density of intramuscular fat cells (*P* > 0.05, Table 5), although the 31% increase in adipocyte density with L-arginine supplementation was almost the same as the increase in IMF, noted above.

**Table 5:** Effect of dietary L-arginine on the diameter and density of adipocytes in longissimus muscle.

Indices	Control	1% arginine
Diameter (μm）	128.14 ± 4.42	132.50 ± 3.42
Density (cells/mm^2^) (*10^2^)	8.09 ± 1.39	10.62 ± 1.85

Data are means ± SEM, n = 24. There were no significant effects of supplementation (*P* < 0.05).

### 5. Effects of L-arginine on plasma biochemical variables

None of the plasma biochemical variables, listed in Table 6, were significantly affected by supplementation with 1% dietary L-arginine (*P* > 0.05).

**Table 6:** Effect of dietary arginine supplementation on blood variables.

Indexes	Control	1% arginine
HDL (mmol/L)	1.08 ± 0.04	1.13 ± 0.05
LDL (mmol/L)	1.05 ± 0.05	1.05 ± 0.05
Cholesterol (mmol/L)	2.15 ± 0.09	2.17 ± 0.08
Triglycerol (mmol/L)	0.45 ± 0.08	0.43 ± 0.07
Glucose (mmol/L)	167.96 ± 7.58	157.13 ± 8.23
Leptin (ng/ml)	0.76 ± 0.12	0.79 ± 0.17

Data are means ± SEM, n = 24. There were no significant effects of supplementation (*P* < 0.05). HDL, high density lipoproteins, LDL, low density lipoproteins

### 6. Effect of dietary L-arginine on proteins of longissimus muscle

**Fig 1:**
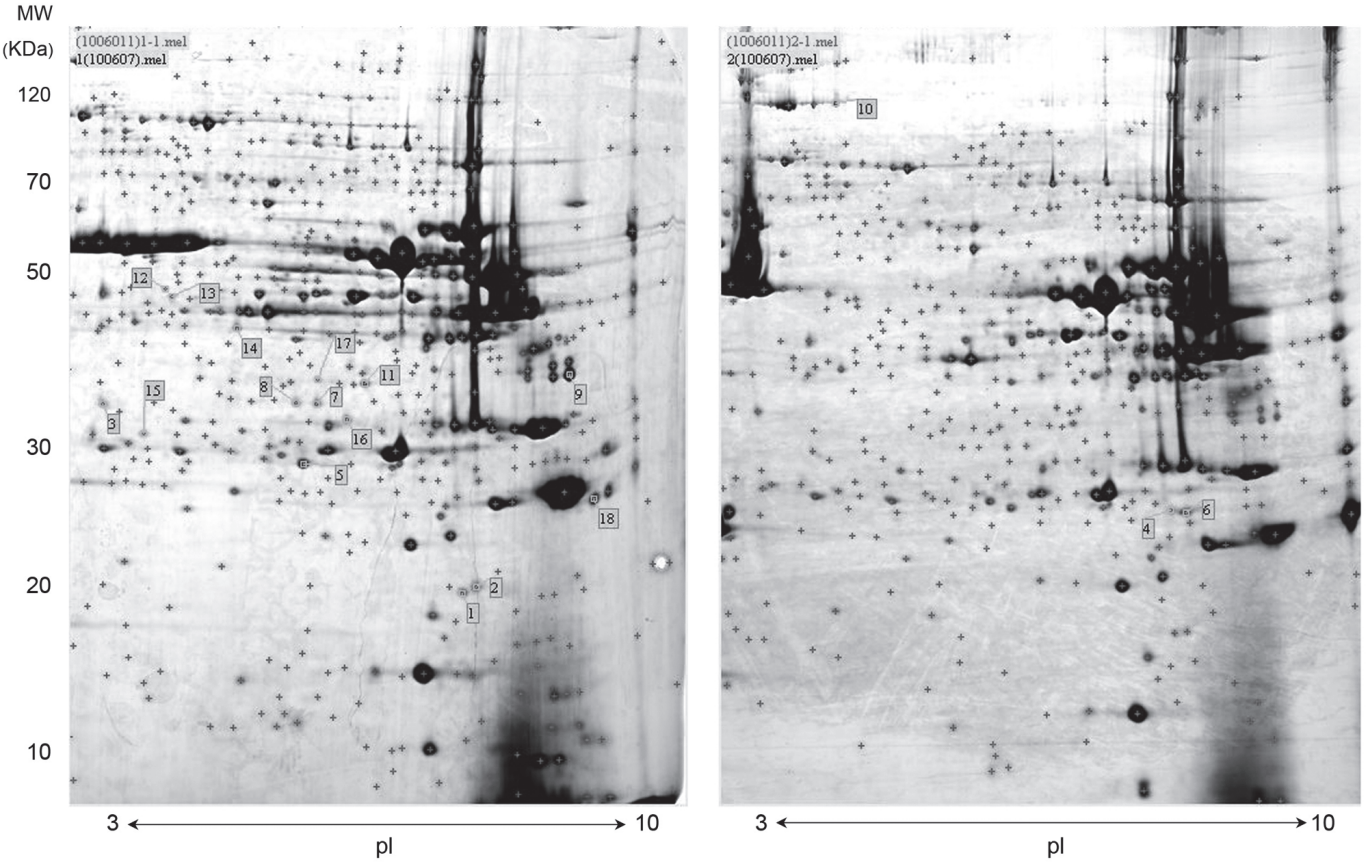
Representative 2-DE images of longissimus muscle samples. 2-DE was per-formed using a pH range of 3–10 in the first dimension and SDS-PAGE (12%) in the second. Control sample is shown at the left and sample from 1% arginine supplementation is shown at the right. Detected protein spots are marked with red “+” and protein abundances that differ significantly between control and supplemented pigs are assigned numbers, corresponding to Table 7. MW, Molecular weight; PI, Isoelectric point.

Using 2-DE, of the approximately 600 proteins detected, 18 were differentially expressed between the controls and arginine supplemented pigs, which were labeled in Fig. 1. The analyzed results are shown in Table 7. Of these 18 proteins, 9 were identified by matching peptide data to porcine protein sequences in the database, and 9 were identified by matching peptide data to human, mouse or sheep (which have interspecies homology of pig) protein sequences in the database (5 to human, 2 to mouse and 2 to sheep). Some identified proteins, including muscle creatine kinase (MCK), TNNI1 and glycogen phosphorylase, were resolved as multiple spots. For example, proteins 1, 2, 5, 11 and 16 were identified as MCK with the same molecular weight (MW) but different isoelectric points (pI); proteins 4 and 6 were identified as slow twitch troponin I protein with the same MW but different pI. According to the proposed functions (http://www.uniprot.org/), the identified proteins conform to 4 groups: structure related (such as α-actin, α-actinin 3, LIM domain binding 3, PDZ and LIM domain7), fiber-type related (such as TNNI1 and MyHC IIb), energy metabolism related (such as muscle glycogen phosphorylase, MCK, cytosolic malate dehydrogenase and muscle-specific enolase β-subunit), and others. Among these protein, TNNI1 and α-actinin 3 were detected only in the arginine-supplemented pigs while the other proteins were significantly down-regulated from the controls.

**Table 7:** Longissimus proteins differentially expressed between control and arginine supplemented pigs.

No^1^	Identified protein	Accession No	Protein MW / pI	Best Ion Score	Intensity matched	Fold Change*	P- value
1	Muscle creatine kinase(*Sus scrofa*)	gi|194018722	43031 / 6.61	47	89.74	0.41	0.015
2	Muscle creatine kinase(*Sus scrofa*)	gi|194018722	43031 / 6.61	52	88.25	0.53	0.001
3	Actin-α1(*Homo sapiens*)	gi|27819614	41919 / 5.30	41	64.50	0.61	0.001
4	Slow-twitch skeletal troponin I (TNNI1) (*Homo sapiens*)	gi|339965	21648 / 9.55	45	67.05	#	
5	Muscle creatine kinase(*Sus scrofa*)	gi|194018722	43031 / 6.61	62	87.68	0.54	0.001
6	Slow-twitch skeletal troponin I (TNNI1) (*Homo sapiens*)	gi|339965	21648 / 9.55	45	67.05	#	
7	Muscle glycogen phosphorylase (*Ovis aries*)	gi|57163939	97245 / 6.65	25	51.79	0.36	0.031
8	Muscle glycogen phosphorylase(*Ovis aries*)	gi|57163939	97245 / 6.65	114	69.03	0.49	0.003
9	LIM domain binding 3(*Homo sapiens*)	gi|122056619	30979 / 9.2	88	46.08	0.42	0.007
10	Actinin alpha 3 (*Mus musculus*)	gi|7304855	102977 / 5.31	31	65.36	#	
11	Muscle creatine kinase(*Sus scrofa*)	gi|194018722	43031 / 6.61	68	83.07	0.55	0.002
12	Phosphoglycerate kinase 1 (*Sus scrofa*)	gi|153792027	44530 / 8.02	30	70.85	0.55	0.001
13	Myosin heavy chain 2b(*Sus scrofa*)	gi|178056718	223097 / 5.6	33	56.65	0.51	0.007
14	Cytosolic malate dehydrogenase (*Sus scrofa*)	gi|47523114	36431 / 6.16	43	77.53	0.49	0.002
15	Peroxiredoxin 6(*Sus scrofa*)	gi|47523870	25021 / 5.73	25	85.38	0.34	0.007
16	Muscle creatine kinase(*Sus scrofa*)	gi|194018722	43031 / 6.61	57	88.38	0.50	0.005
17	Muscle-specific enolase β-subunit (*Homo sapiens*)	gi|34789	46929 / 7.59	51	51.56	0.47	0.003
18	PDZ and LIM domain7 *[Mus musculus]*	gi|29436855	21083 / 9.66	61	58.01	0.41	0.001

^1^ corresponding to numbers in Fig. 1,

* L-arginine supplemented/control,

# only detected in the supplemented samples.

**Fig 2:**
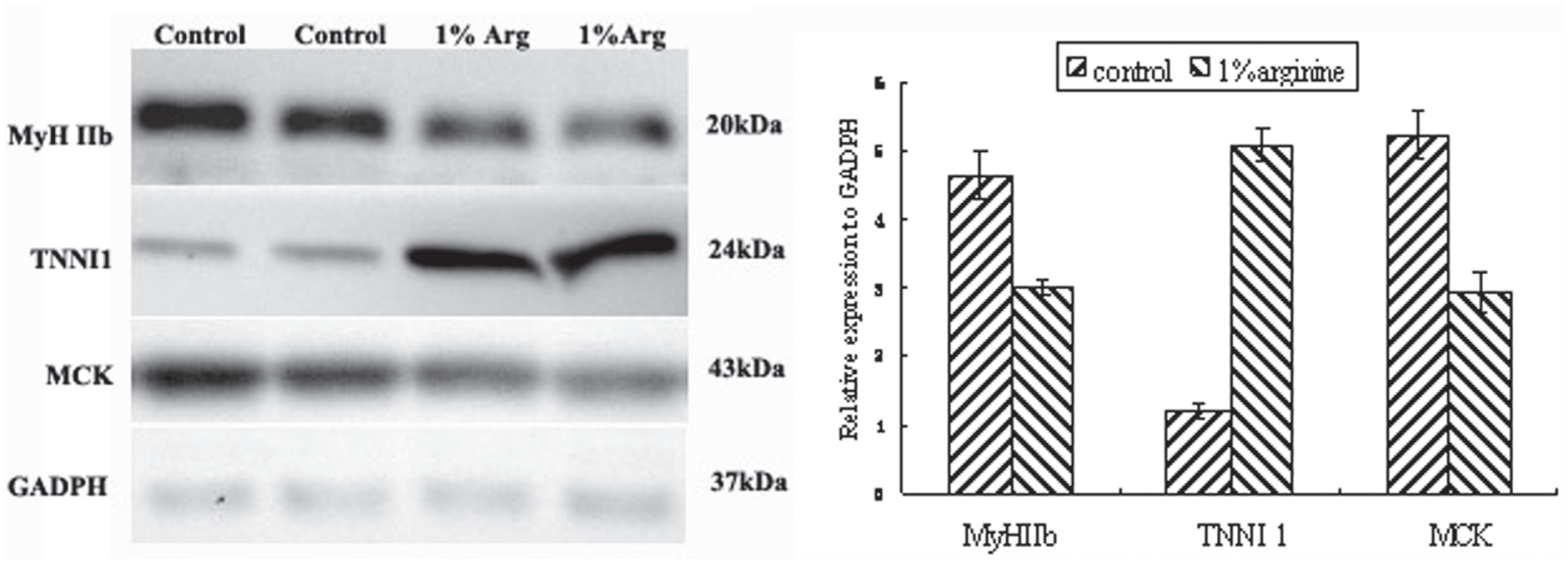
Western-blots for a selection of differentially-expressed proteins. MyH2b, TNNI1 and MCK were used as target protein; GADPH was used as a control. A: Total protein from control and arginine-supplemented pig samples was fractionated by SDS-PAGE, blotted and then detected with specific antibodies; B: Semi-quantification of protein content computed using Image J software. Data are presented as the mean ±SEM (n = 6).

To confirm the differential expression between the controls and supplemented pigs, 3 proteins (TNNI1, MCK and MyHC IIb) were quantified by Western blotting (Fig. 2). There was much more TNNI1 in muscle from L-arginine supplemented pigs than in the controls, whereas MyHC IIb and MCK were more abundant in the controls than in the L-arginine supplemented pigs.

## Discussion

L-Arginine has been known to reduce excessive amounts of white adipose tissue [39] and increase lean tissue mass [1] in animals through multiple signaling pathways [40]. Notably, dietary supplementation with arginine enhances intramuscular fat content in finishing pigs and improves meat quality [4] in association with alterations of fatty acid composition in white adipose tissue [20]. As a functional amino acid, arginine regulates protein and lipid metabolism in a tissue-specific manner [41]. Results of the proteome changes in skeletal muscle provide further insight into mechanisms responsible for beneficial effects of arginine supplementation on nutrient metabolism and meat quality of swine.

### 1. Effect of dietary L-arginine on intramuscular fat deposition

The present experiment confirmed an earlier finding [5] that dietary supplementation of pigs with 1% L-arginine increased IMF content by about 32%. It also was found that the L-arginine caused a similar, though non-significant, increase in the density of intramuscular fat cells. In addition, L-arginine also increased the relative content of total PUFA, but did not affect the relative content of total SFA and MUFA in the extracted lipids. Cameron et al. [42] showed that the proportions of C18:2, C20:4 and C22:6 polyunsaturated fatty acids were positively correlated with meat flavor. Yu et al.[43] found that Lantang pigs had a higher C18:2, C20:4 and C22:6, showing better pork flavor intensity, which supported the results of Cameron et al. [42]. Though flavor was not assessed in the present experiment, L-arginine increased the relative contents of C20:1 (32%) and C22:6 (153%), which may be related to flavor enhancement and good for human health [44].

### 2. Dietary L-arginine affected the proteome profile of muscle

**Structure-related proteins.** Four structure-related proteins were differentially expressed; α-actin, α-actinin 3, LIM domain binding 3, PDZ and LIM domain7 isoform 1 were all reduced in pigs supplemented with L-arginine. The highly conserved α-actin is associated with myofibrils and muscle development [45]. The IMF content in Lantang pigs, a Chinese obese-type breed, is higher than in Landrace while the α-actin content in longissimus is reversed, Landrace/Lantang being about 1.6, which implied that IMF content is negatively correlated with α- actin [46]. In steers, the α- actin and β- actin protein content also decreased as IMF increased during the fattening stage [47]. Some reports also showed that the expression of actin and several other cytoskeletal proteins was down-regulated during adipogenesis [48–49]. Taken together, actin protein, involving cell junction, might have negative effect of IMF accumulation. The present results are consistent with others’ findings that dietary arginine stimulated IMF deposition, probably by decreasing the content of α-actin, so further study is warranted on the reason why arginine supplementation decreases the muscle content of α-actin. The Z-disc structural protein, α-actinin-3, is found only in type II muscle fibers, and especially in type IIx [50]. Broos et al. [51] reported that the lack of α-actinin-3 in a mouse model resulted in less stiff type II (a)/II(x) fibers and decreased unloaded shortening velocity and increased fiber elasticity. Based on our result showing increased α-actinin-3 in 1% L-arginine supplemented pigs, we speculate that L-arginine decreased the proportion of type II (b) fibers in muscle. We also found that myosin heavy chain 2b (MyHC IIb) decreased significantly in the L-arginine supplemented pigs. PDZ-LIM protein is the α-actinin-associated LIM protein (ALP). Over-expression of LIM kinase enhanced actin stability [52–53]. The PDZ domain of Pdlim3, Pdlim5 (also known as ENH) and LMO7 binds to α-actinin at adherens junctions [54] and improved the function of α-actin. Our result showed that the content of these two proteins in muscle decreased in the L-arginine supplemented pigs, which may be one of the reasons underlying the increase in intramuscular fat content.

**Proteins related to Fiber type.** Two proteins related to fiber type were identified: TNNI1 and MyHC IIb. The former is expressed in oxidative fibers (slow-twitch skeletal muscle isoform) [55–56] and has been used as a model gene to study mechanisms of slow fiber-specific expression [57]. Muscle with a higher content of oxidative fibers contains a higher percentage of lipids [58]. The flavor, tenderness or IMF content also was associated with a higher content of oxidative fibers in muscles [59]. Yang et al. [60] found that the TNNI1 gene polymorphism was associated with the content of IMF. Pierzchala et al.[61] also found that TNNI1 gene was associated with meat quality. Recently, Xu et al. [62] found that the content of IMF and TNNI1 were higher in Meishan pigs than in Yorkshire pigs, while the content of MyHC IIb was the reverse. MyHC IIb was specially expressed in glycolic (type IIb) fibers, which contain greater amounts of glycogen and glucose, and predominantly use them as fuel [63]. Muroya et al [64] reported that proteolysis in type IIb has greater rate and extent than in type I fibers and TNNI1 protein is higher in type I fibers and degraded slower than TNNI2 protein in porcine longissimus muscle. The present study found that supplemental arginine increased IMF and TNNI1 contents, while decreasing that of MyHC IIb, supporting IMF content being positively associated with TNNI1 and negatively with MyHC IIb [19].All of this leads to deducing that arginine increased IMF content by changing the fiber type makeup of the muscle.

**Energy metabolism.** The proteins related to energy metabolism were identified as being affected by arginine supplementation: muscle creatine kinase, glycogen phosphorylase, phosphoglycerate kinase 1, cytosolic malate dehydrogenase and muscle-specific enolase beta subunit. Muscle creatine kinase (MCK) is among the most abundant transcripts in striated muscle, and the protein as well as its enzymatic product, creatine phosphate, was about 2 to 3 times higher in fast-twitch muscles than in slow twitch muscles [65–66]. Here, we found that MCK was reduced by dietary L-arginine supplementation, and the slow twitch muscle indices were increased, relative the controls, consistent with the results of Kushmerick et al. [65] and Yamashita et al. [66]. Glycogen phosphorylase, phosphoglycerate kinase 1 and muscle-specific enolase beta subunit are key glycolytic enzymes [67]. Comparisons between wild and domestic pigs [68] showed that intensive selection for lean muscle growth in modern pigs has induced a shift in muscle metabolism toward a more glycolytic and less oxidative fiber types. Fast-twitch glycolytic fibers, also referred to as white fibers, contained high quantities of glycogen [69], and inhibition of glycogen phosphorylase leads to reduced glycogen degradation [70]. Guo et al. [58] also reported that Jinhua pigs possessed a lower rate of glycolysis and a higher rate of oxidative metabolism compared with Landrace pigs (with less intramuscular fat). Our result showed that these enzymes decreased in L-arginine supplemented pigs, which implied reduced capacity for glycogen metabolism than in the control pigs. There is little known about mRNA expression or protein content of peroxiredoxin 6 (PRDX6) in pigs, but Keady et al. [71] reported that PRDX6 in steer muscle may be related to lipid catabolism. The function of this protein in pigs needs further study.

In conclusion, the differentially expressed muscle fiber types and metabolic enzymes resulting from arginine supplementation may be important factors influencing meat quality, particularly IMF content and drip loss, in the pigs examined here. These results provide valuable information for better understanding of the mechanism by which supplemental L-arginine improves meat quality by increasing IMF content and fiber type transformation, and may provide a basis for manipulating muscle fiber type to improve meat quality.
